# Serum ferritin in combination with prostate-specific antigen improves predictive accuracy for prostate cancer

**DOI:** 10.18632/oncotarget.14977

**Published:** 2017-02-01

**Authors:** Xijuan Wang, Peng An, Jiling Zeng, Xiaoyan Liu, Bo Wang, Xuexian Fang, Fudi Wang, Guoping Ren, Junxia Min

**Affiliations:** ^1^ The First Affiliated Hospital, Institute of Translational Medicine, School of Public Health, Collaborative Innovation Center for Diagnosis and Treatment of Infectious Diseases, School of Medicine, Zhejiang University, Hangzhou 310058, China; ^2^ Department of Nutrition, Precision Nutrition Innovation Center, School of Public Health, Zhengzhou University, Zhengzhou 450001, China

**Keywords:** prostate cancer, ferritin, PSA

## Abstract

Ferritin is highly expressed in many cancer types. Although a few studies have reported an association between high serum ferritin levels and an increased risk of prostate cancer, the results are inconsistent. Therefore, we performed a large case-control study consisting of 2002 prostate cancer patients and 951 control patients with benign prostatic hyperplasia (BPH). We found that high ferritin levels were positively associated with increased serum prostate-specific antigen (PSA) levels and prostate cancer risk; each 100 ng/ml increase in serum ferritin increased the odds ratio (OR) by 1.20 (95% CI: 1.13−1.36). In the prostate cancer group, increased serum ferritin levels were significantly correlated with higher Gleason scores (*p* < 0.001). Notably, serum PSA values had even higher predictive accuracy among prostate cancer patients with serum ferritin levels > 400 ng/ml (Gleason score + total PSA correlation: *r* = 0.38; Gleason score + free PSA correlation: *r* = 0.49). Moreover, using immunohistochemistry, we found that prostate tissue ferritin levels were significantly higher (*p* < 0.001) in prostate cancer patients *(n* = 129) compared to BPH controls (*n* = 31). Prostate tissue ferritin levels were also highly correlated with serum ferritin when patients were classified by cancer severity (*r* = 0.81). Importantly, we found no correlation between serum ferritin levels and the inflammation marker C-reactive protein (CRP) in prostate cancer patients. In conclusion, serum ferritin is significantly associated with prostate cancer and may serve as a non-invasive biomarker to complement the PSA test in the diagnosis and prognostic evaluation of prostate cancer.

## INTRODUCTION

Prostate cancer is one of the most common malignancies among men and is the third leading cause of cancer-related deaths among men [1]. Prostate-specific antigen (PSA) has been widely used as a clinical diagnostic biomarker for prostate cancer [2]. However, PSA is not necessarily specific for prostate cancer, and elevated PSA levels have also been reported in patients with benign prostatic hyperplasia (BPH), prostatitis, and following physical trauma to the prostate (e.g., bicycling injury, digital rectal examination, catheterization, etc.) [3, 4]. Moreover, prostate cancer patients can have PSA levels ≤ 4 ng/ml, which is generally considered to be in the “normal” range, thereby contributing to misdiagnosis [5]. Thus, PSA may not be the most suitable biomarker for prostate cancer, given its general lack of specificity and sensitivity [6]. To increase diagnostic accuracy and reduce the number of unnecessary screening procedures, biopsies, and treatments [6, 7], new and/or complementary non-invasive biomarkers for prostate cancer are needed.

In the body, ferritin is the primary iron storage protein. Thus, ferritin plays roles in many physiological and pathological processes, including immunosuppression [8], proliferation [9], angiogenesis [10], and carcinogenesis [11]. Moreover, an increasing number of studies have demonstrated that circulating ferritin is elevated in patients with a variety of malignant cancer types [12–18] and is associated with cancer risk [19–21]. In addition, recent studies have suggested the potential clinical value of circulating ferritin in several malignancies, including hepatocellular carcinoma [13], lung carcinoma [14], non-Hodgkin lymphoma [16], pancreatic cancer [22], and colorectal cancer [23]. These studies support the notion that ferritin may serve as a suitable biomarker in the diagnosis and prognosis of various cancer types.

Despite previous research showing a possible correlation between ferritin and various cancer types, there is limited evidence regarding the putative relationship between ferritin and the risk of prostate cancer. Therefore, we conducted a large-scale case-control study in order to examine the relationship between serum ferritin and the risk, diagnosis, and prognosis associated with prostate cancer.

## RESULTS

### Prostate cancer patients have significantly higher serum ferritin levels compared to BPH (control) patients

Compared to control patients with benign prostatic hyperplasia (BPH), the patients with prostate cancer had higher levels of total PSA and free PSA, as well as a lower ratio of free PSA to total PSA (Table 1). Moreover, the prostate cancer group contained significantly higher percentages of patients with high serum ferritin levels (i.e., > 300, > 400, and > 500 ng/ml; *p* < 0.001 for all three categories); these results are summarized in Table 1.

**Table 1:** Patient cohort characteristics

	Benign prostatic hyperplasia	Prostate cancer	*P*-value
*n*	951	2002	
Age, years	69.75 (8.37)	70.13 (7.80)	0.23
Total PSA, ng/ml	4.12 (5.95)	16.05 (33.10)	< 0.001
Free PSA, ng/ml	0.81 (1.05)	1.44 (2.20)	< 0.001
Free PSA/Total PSA ratio	0.23 (0.25)	0.12 (0.08)	< 0.001
Serum ferritin, ng/ml	180.80 (150.45)	200.85 (176.35)	< 0.001
Hyperferritinemia			
Serum ferritin > 300 ng/ml, *n* (%)	181 (19.03)	517 (25.82)	< 0.001
Serum ferritin > 400 ng/ml, *n* (%)	60 (6.31)	269 (13.44)	< 0.001
Serum ferritin > 500 ng/ml, *n* (%)	32 (3.36)	149 (7.44)	< 0.001
Hemoglobin, g/L	139.10 (15.63)	139.70 (17.51)	0.41
Triglycerides, mmol/L	1.28 (0.67)	1.42 (0.89)	< 0.001
Total cholesterol, mmol/L	4.39 (0.87)	4.47 (0.89)	0.007
HDL cholesterol, mmol/L	1.17 (0.34)	1.17 (0.33)	0.67
LDL cholesterol, mmol/L	2.41 (0.65)	2.51 (0.67)	< 0.001
VLDL cholesterol, mmol/L	0.82 (0.39)	0.83 (1.29)	0.04
Dyslipidemia, *n* (%)	543 (57.10)	1189 (59.39)	0.33
Systolic blood pressure, mmHg	131.07 (16.78)	130.52 (15.68)	0.55
Diastolic blood pressure, mmHg	78.34 (10.20)	77.62 (10.00)	0.04
Hypertension, *n* (%)	409 (43.01)	895 (44.71)	0.61
Diabetes, *n* (%)	119 (12.51)	245 (12.24)	0.90
ALT, U/L	20.52 (12.39)	21.72 (19.71)	0.51
AST, U/L	21.74 (7.28)	22.58 (9.63)	0.06
GGT, U/L	31.28 (30.30)	35.81 (39.65)	< 0.001
DBIL, μmol/L	4.12 (2.09)	4.12 (1.91)	0.42
IBIL, μmol/L	8.34 (3.63)	8.25 (3.94)	0.16
Uric acid, μmol/L	352.60 (82.74)	360.95 (90.31)	0.17
Drinking			0.84
Nondrinkers, *n* (%)	642 (67.51)	1343 (67.08)	
Previous drinkers, *n* (%)	74 (7.78)	145 (7.24)	
Current drinkers, *n* (%)	233 (24.50)	502 (25.07)	
Smoking			0.30
Non-smokers, *n* (%)	565 (59.41)	1193 (59.59)	
Previous smokers, *n* (%)	122 (12.83)	290 (14.49)	
Current smokers, *n* (%)	262 (27.55)	508 (25.37)	
Education, years			< 0.001
0–6, *n* (%)	469 (49.32)	745 (37.21)	
7–12, *n* (%)	349 (36.70)	851 (42.51)	
> 12, *n* (%)	118 (12.41)	383 (19.13)	

Notes: Unless indicated otherwise, data are presented as the median value (IQR). The Wilcoxon rank-sum test was used for comparing two groups, and the Pearson's chi-square test was used for categorical data. PSA, prostate-specific antigen; HDL, high-density lipoprotein; LDL, low-density lipoprotein; VLDL, very low-density lipoprotein; ALT, alanine transaminase; AST, aspartate transaminase; GGT, gamma-glutamyl transferase; DBIL, direct bilirubin; IBIL, indirect bilirubin.

### High serum ferritin levels in prostate cancer patients are associated with TNM classification

The clinicopathological data and statistical analyses for the patients in the prostate cancer group are summarized in Table 2. Our analysis revealed that Gleason score and serum total PSA values differed significantly when the patients were classified according to pathological tumor stage, lymph node involvement, and distant metastasis. These findings suggest a possible correlation between TNM (tumor, node, metastasis) classification and serum ferritin levels.

**Table 2:** Characteristics of prostate cancer patients

	n	Gleason score			*P*-value ^b^	Serum total PSA (ng/ml)				*P*-value ^b^	Serum ferritin (ng/ml)		*P*-value ^b^	Serum ferritin (ng/ml)		*P*-value ^b^	Serum ferritin (ng/ml)		*P*-value ^b^
		≤ 6	7	≥ 8		< 4	4-9.9	10–20	> 20		≤ 300	> 300		≤ 400	> 400		≤ 500	> 500	
Pathological tumor stage ^a^	1101				< 0.001					< 0.001			0.745			0.025			0.656
pT2	778 (70.7%)	171	482	125		97	265	254	162		597	181		697	81		737	41	
pT3	292 (26.5%)	9	148	135		31	39	79	143		225	67		254	38		273	19	
pT4	31 (2.8%)	0	4	27		2	2	3	24		22	9		23	8		29	2	
Lymph node involvement ^a^	867				< 0.001					< 0.001			< 0.001			< 0.001			< 0.001
N0	720 (83%)	107	444	169		77	189	252	202		565	155		651	69		688	32	
N1	147 (17%)	3	34	110		7	11	15	114		87	60		112	35		127	20	
Distant metastasis ^a^	504				< 0.001					< 0.001			< 0.001			< 0.001			< 0.001
M0	354 (70.2%)	54	223	77		37	91	95	131		270	84		313	41		335	19	
M1	150 (29.8%)	2	26	122		3	6	7	134		76	74		99	51		113	37	
Percent positive biopsy cores ^a^	578				< 0.001					< 0.001			0.109			< 0.001			0.009
0–25%	143 (24.8%)	60	71	12		9	51	55	28		115	28		137	6		139	4	
26–50%	158 (27.3%)	22	87	49		5	44	52	57		118	40		135	23		145	13	
51–75%	119 (20.6%)	5	66	48		5	17	33	64		85	34		98	21		108	11	
76–100%	158 (27.3%)	2	58	98		3	7	19	129		108	50		125	33		137	21	
Therapy ^a^	1250				< 0.001					< 0.001			< 0.001			< 0.001			0.106
Radical prostatectomy	996 (79.7%)	186	613	197		117	307	315	257		225	771		893	103		174	51	
Radiation therapy	106 (8.5%)	36	41	29		4	29	26	47		82	24		98	8		21	3	
Hormonal therapy	148 (11.8%)	7	47	94		2	10	20	116		91	57		118	30		38	19	

Notes: N0, tumor cells absent from regional lymph nodes; N1, regional lymph node metastasis present; M0, no distant metastasis; M1, metastasis to distant organs (beyond regional lymph nodes).

^a^ Data were not available for all 2002 prostate cancer patients. ^b^ Pearson's chi-square test was used to calculate the *p*-values.

### High serum ferritin is associated with increased serum PSA levels and increased prostate cancer risk

Next, we analyzed the association between serum ferritin, serum PSA levels, and prostate cancer risk; these results are summarized in Table 3. We found that high serum ferritin was significantly associated with increased serum total PSA levels (*p* < 0.001). In addition, increased serum ferritin was associated with increased prostate cancer risk; for each 100 ng/ml increase in serum ferritin, the odds ratio (OR) for prostate cancer risk was 1.20 (95% CI: 1.13–1.36) (*p* < 0.001). Consistent with these results, high serum ferritin (defined as >300 ng/ml) was also significantly associated with increased total PSA levels and prostate cancer risk (OR: 1.52; 95% CI: 1.25–1.85) (*p* < 0.001).

**Table 3:** Association between various serum parameters and serum PSA values and prostate cancer risk

	Total PSA ^a^		Free PSA ^a^		Free PSA/Total PSA ratio ^a^		Prostate cancer risk	
	beta (s.e.)	*P*-value	beta (s.e.)	*P*-value	beta (s.e.)	*P*-value	OR (95% CI)	*P*-value
Serum ferritin, 100 ng/ml	0.12 (0.01)	< 0.001	0.04 (0.01)	< 0.001	−0.01 (0.007)	0.13	1.20 (1.13–1.26)	< 0.001
Hyperferritinemia	0.39 (0.08)	< 0.001	0.05 (0.06)	0.43	−0.06 (0.03)	0.07	1.52 (1.25–1.85)	< 0.001
Hemoglobin, g/L	−0.006 (0.002)	0.003	0.001 (0.001)	0.47	−0.002 (0.0008)	0.03	1.00 (0.996–1.01)	0.67
Triglycerides, mmol/L ^a^	0.06 (0.07)	0.39	0.06 (0.05)	0.23	−0.07 (0.03)	0.01	1.52 (1.28–1.81)	< 0.001
Total cholesterol, mmol/L	−0.03 (0.04)	0.43	0.04 (0.03)	0.13	−0.02 (0.01)	0.23	1.11 (1.01–1.21)	0.03
HDL cholesterol, mmol/L	−0.13 (0.10)	0.195	−0.05 (0.08)	0.49	0.11 (0.04)	0.008	1.04 (0.82–1.31)	0.76
LDL cholesterol, mmol/L	−0.009 (0.05)	0.87	0.04 (0.04)	0.21	−0.03 (0.02)	0.13	1.25 (1.11–1.41)	< 0.001
VLDL cholesterol, mmol/L	0.02 (0.03)	0.54	0.03 (0.02)	0.15	−0.009 (0.01)	0.41	1.01 (0.93–1.09)	0.80
ALT, U/L ^a^	−0.17 (0.07)	0.01	−0.04 (0.05)	0.35	−0.02 (0.03)	0.34	1.11 (0.95–1.30)	0.18
AST, U/L ^a^	0.09 (0.11)	0.44	−0.05 (0.08)	0.51	−0.01 (0.04)	0.78	1.38 (1.07–1.79)	0.01
GGT, U/L ^a^	0.25 (0.06)	< 0.001	0.04 (0.04)	0.37	−0.04 (0.02)	0.09	1.31 (1.15–1.50)	< 0.001
DBIL, μmol/L ^a^	0.06 (0.08)	0.47	0.03 (0.05)	0.59	0.01 (0.03)	0.66	1.12 (0.94–1.34)	0.20
IBIL, μmol/L ^a^	−0.095 (0.07)	0.19	0.008 (0.05)	0.87	0.04 (0.03)	0.11	0.89 (0.75–1.05)	0.16
Uric acid, μmol/L	0.001 (0.0005)	0.02	0.0004 (0.0004)	0.31	0.00008 (0.0002)	0.66	1.00 (0.9995–1.00)	0.22
Systolic blood pressure, mmHg	−0.0007 (0.002)	0.76	0.002 (0.002)	0.15	0.001 (0.0008)	0.11	1.00 (0.99–1.00)	0.19
Diastolic blood pressure, mmHg	−0.007 (0.003)	0.05	0.0006 (0.002)	0.79	0.003 (0.001)	0.04	0.99 (0.99–1.00)	0.10

Notes: The data were obtained using either a linear regression model (with serum PSA as the dependent variable) or a logistic regression model adjusted for age, education level, smoking category, drinking category, and diabetes status. PSA, prostate-specific antigen; HDL, high-density lipoprotein; LDL, low-density lipoprotein; VLDL, very low-density lipoprotein; ALT, alanine transaminase; AST, aspartate transaminase; GGT, gamma-glutamyl transferase; DBIL, direct bilirubin; IBIL, indirect bilirubin; OR, odds ratio.

^a^ Natural log-transformed values in regression analysis.

### Serum ferritin in diagnosing prostate cancer

**Figure 1:**
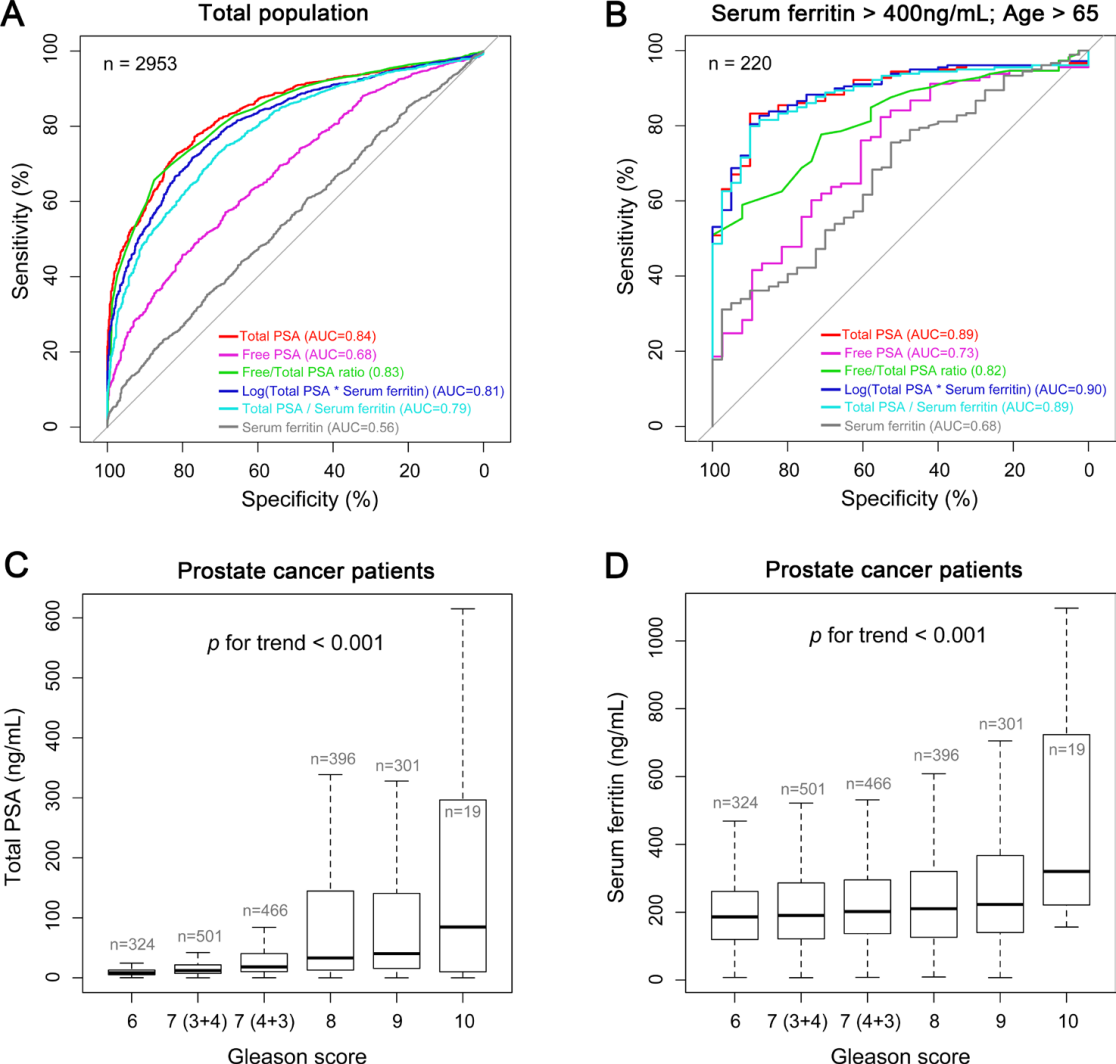
Serum ferritin and PSA levels in prostate cancer and control patients (**A** and **B**) The sensitivity and specificity of serum ferritin were analyzed using receiver operating characteristic (ROC) curves in the total study population (both the prostate cancer and BPH groups) and the elderly subpopulation with hyperferritinemia (serum ferritin > 400 ng/ml and > 65 years of age). For each measurement, the area under the curve (AUC) is indicated. (**C** and **D**) Serum total PSA (C) and serum ferritin levels (D) were measured in the prostate cancer patients and are plotted according to Gleason score.

To access whether serum ferritin can be used as either a primary or secondary diagnostic biomarker for prostate cancer, we measured the sensitivity and specificity of serum ferritin in prostate cancer using receiver operating characteristic (ROC) curve analysis. Within the entire study cohort (i.e., the BPH and prostate cancer groups combined), serum total PSA and the free PSA/total PSA ratio yielded the largest area under the curve (AUC) values (0.84 and 0.83, respectively; Figure 1A). When serum ferritin was used either alone or together with serum total PSA, the AUC was smaller than the AUC for either total PSA or the free PSA/total PSA ratio, indicating that serum ferritin is not a sufficiently sensitive biomarker at the total cohort level. As shown in Table 1, the percentage of patients with hyperferritinemia (defined as serum ferritin > 300 ng/ml) was significantly higher in the prostate cancer group than in the BPH group, particularly among patients with serum ferritin > 400 ng/ml. When we analyzed only patients > 65 years of age with a serum ferritin level > 400 ng/ml, we found increased sensitivity in terms of diagnosing prostate cancer using total PSA (AUC = 0.89) or [log (total PSA)*(serum ferritin)] (AUC = 0.90) (Figure 1B). This indicates that serum ferritin alone may not be a sufficiently sensitive indicator for prostate cancer, but can be useful as a cut-off value for diagnosing elderly patients with hyperferritinemia.

### Serum ferritin is correlated with prognostic indicators

Next, we analyzed the prognostic value of total PSA and serum ferritin in prostate cancer patients by plotting these values against Gleason score. Both serum total PSA levels (Figure 1C) and serum ferritin levels (Figure 1D) increased significantly as Gleason score increased (*p* < 0.001). These data suggest that serum ferritin may serve as a prognostic marker in patients with prostate cancer.

### Improved predicative accuracy of serum PSA for patients with hyperferritinemia

Next, we investigated further whether serum ferritin might be valuable as a prognostic marker for prostate cancer. In the BPH group, serum ferritin was not significantly correlated with total PSA, free PSA, or free PSA/total PSA ratio (Table 4). In contrast, we found significant correlations between both total PSA and free PSA in the prostate cancer group (Table 4). Within the entire prostate cancer group, the correlation coefficient (r) between total PSA and Gleason score was 0.30, and the correlation coefficient between free PSA and Gleason score was 0.33 (Table 4). When we selectively analyzed the prostate cancer patients with serum ferritin > 400 ng/ml, these correlation coefficients increased to 0.38 and 0.49, respectively (Table 4). These results indicate that total and free PSA levels are a more robust indicator of cancer prognosis in patients with hyperferritinemia.

**Table 4:** Improved prognosis accuracy using serum PSA for patients with hyperferritinemia

	BPH		PCa		PCa & ferritin > 300 ng/ml		PCa & ferritin > 400 ng/ml	
	*r*	*P* value	*r*	*P* value	*r*	*P* value	*r*	*P* value
Serum ferritin ~ Total PSA	0.04	0.22	0.29	< 0.001	0.34	< 0.001	0.30	< 0.001
Serum ferritin ~ Free PSA	0.04	0.22	0.18	< 0.001	0.29	< 0.001	0.35	< 0.001
Serum ferritin ~ Free/Total PSA ratio	0.06	0.11	0.03	0.28	0.07	0.15	0.06	0.43
Total PSA ~ Gleason score	−	−	0.30	< 0.001	0.35	< 0.001	0.38	< 0.001
Free PSA ~ Gleason score	−	−	0.33	< 0.001	0.41	< 0.001	0.49	< 0.001
Free/Total PSA ratio ~ Gleason score	−	−	−0.05	0.04	0.13	0.01	0.16	0.03

BPH, benign prostatic hyperplasia patients (*n* = 951); PCa, prostate cancer patients (*n* = 2002); PCa & ferritin > 300 ng/ml, prostate cancer patients with serum ferritin levels > 300 ng/ml (*n* = 509); PCa & ferritin > 400 ng/ml, prostate cancer patients with serum ferritin levels > 400 ng/ml (*n* = 261). Serum ferritin was log-transformed value.

### High levels of prostate tissue ferritin indicate poor prognosis

**Figure 2:**
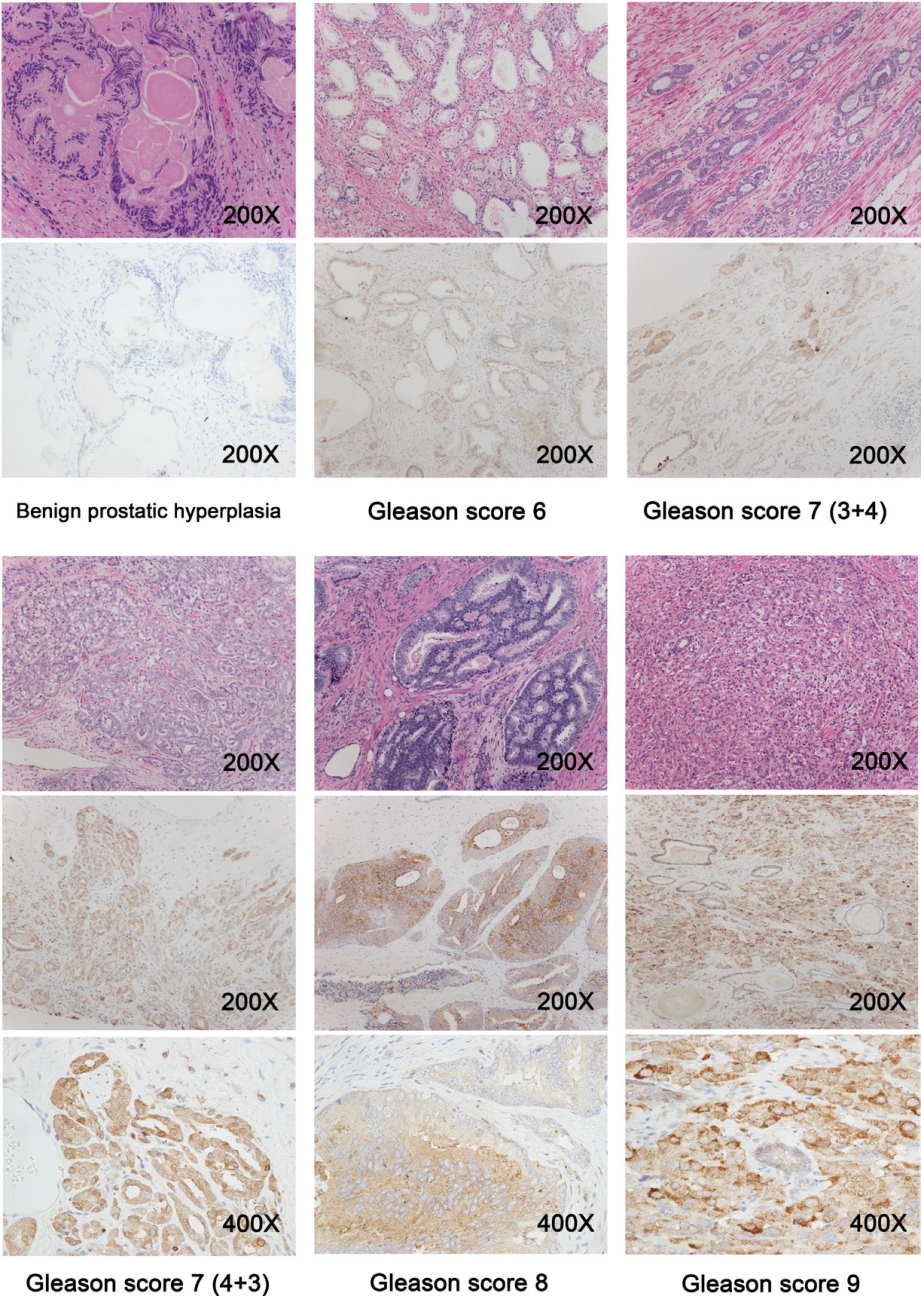
Prostate tissue sections were obtained from benign prostatic hyperplasia patients (*n* = 31) and prostate cancer patients with a Gleason score of 6 to 9 (*n* = 129), and representative immunohistochemistry images are shown

**Figure 3:**
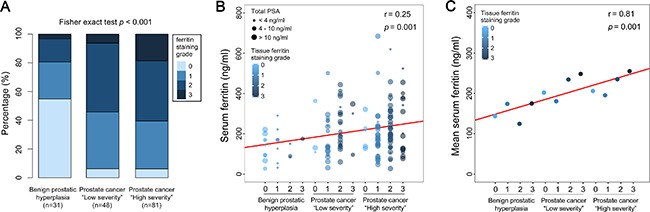
High levels of prostate tissue ferritin are correlated with high clinical severity in prostate cancer patients Prostate tissue sections obtained from BPH patients (*n* = 31) and prostate cancer patients with Gleason scores ranging from 6 to 9 (*n* = 129) were immunostained for ferritin; each section was then graded from 0 to 3 based on the signal intensity. The prostate cancer patients were categorized as “low severity” (*n* = 48) or “high severity” (*n* = 81) based on a combination of serum PSA levels, Gleason score, and TNM classification, in accordance with the 2015 NCCN guidelines. “Low severity” patients were defined as follows: Gleason score ≤ 7, tumor stage ≤ T2c, and PSA ≤ 20 ng/ml; “high severity” patients were defined having as one or more of the following criteria: Gleason score ≥ 8, tumor stage ≥ T3a, and/or PSA > 20 ng/ml). (**A**) Distribution of grade 0, 1, 2, and 3 sections in the BPH, “low severity” prostate cancer, and “high severity” prostate cancer groups. (**B**) Bubble plot showing serum ferritin, total PSA, and tissue ferritin staining grade for each patient in the BPH, “low severity” prostate cancer, and “high severity” prostate cancer groups; the size of each bubble represents total PSA concentration. The solid red line represents the linear fit of the data (*r* = 0.25; *p* = 0.001). (**C**) Mean serum ferritin levels in the indicated immunostaining grades are plotted for each group. The solid red line represents the linear fit of the data (*r* = 0.81; *p* = 0.001).

To investigate the relationship between ferritin levels in the prostate tissue and prognostic outcome, we immunostained prostate tissue sections obtained from BPH patients (*n* = 31) and prostate cancer patients with a Gleason score of 6–9 (*n* = 129). Representative immunohistochemistry images are shown in Figure 2. Based on the intensity of ferritin immunostaining, we assigned a staining grade (ranging from 0 to 3) to each prostate tissue section. We also divided the prostate cancer patients into “low severity” (*n* = 48) and “high severity” (*n* = 81) subgroups using a modified combination of Gleason score, TNM classification, and PSA levels in accordance with the 2015 NCCN (National Comprehensive Cancer Network) guidelines. We found that the distribution of ferritin immunostaining grade differed significantly among the BPH, “low severity”, and “high severity” groups (*p* < 0.001; Figure 3A). Specifically, the sections with a strong positive ferritin signal (i.e., grade 3 staining) were more common in the “high severity” group than in the “low severity” group; moreover, 17 out of 31 sections in the BPH group had no ferritin signal (grade 0 staining). For each patient in each group (i.e., BPH, “low severity” prostate cancer, and “high severity prostate cancer”), the corresponding serum ferritin level was then calculated and plotted against tissue ferritin staining grade (0, 1, 2, or 3) in a bubble plot, with the size of each bubble corresponding to that patient's total PSA level (Figure 3B). Our analysis revealed that serum ferritin levels were significantly correlated with tissue ferritin levels (*r* = 0.25; *p* = 0.001). Plotting the mean serum ferritin levels in each grade within each patient group yielded an even stronger correlation (*r* = 0.81; *p* = 0.001) between serum ferritin levels and tissue ferritin levels (Figure 3C).

### Serum ferritin level is not correlated with the inflammation marker CRP in prostate cancer patients

**Figure 4:**
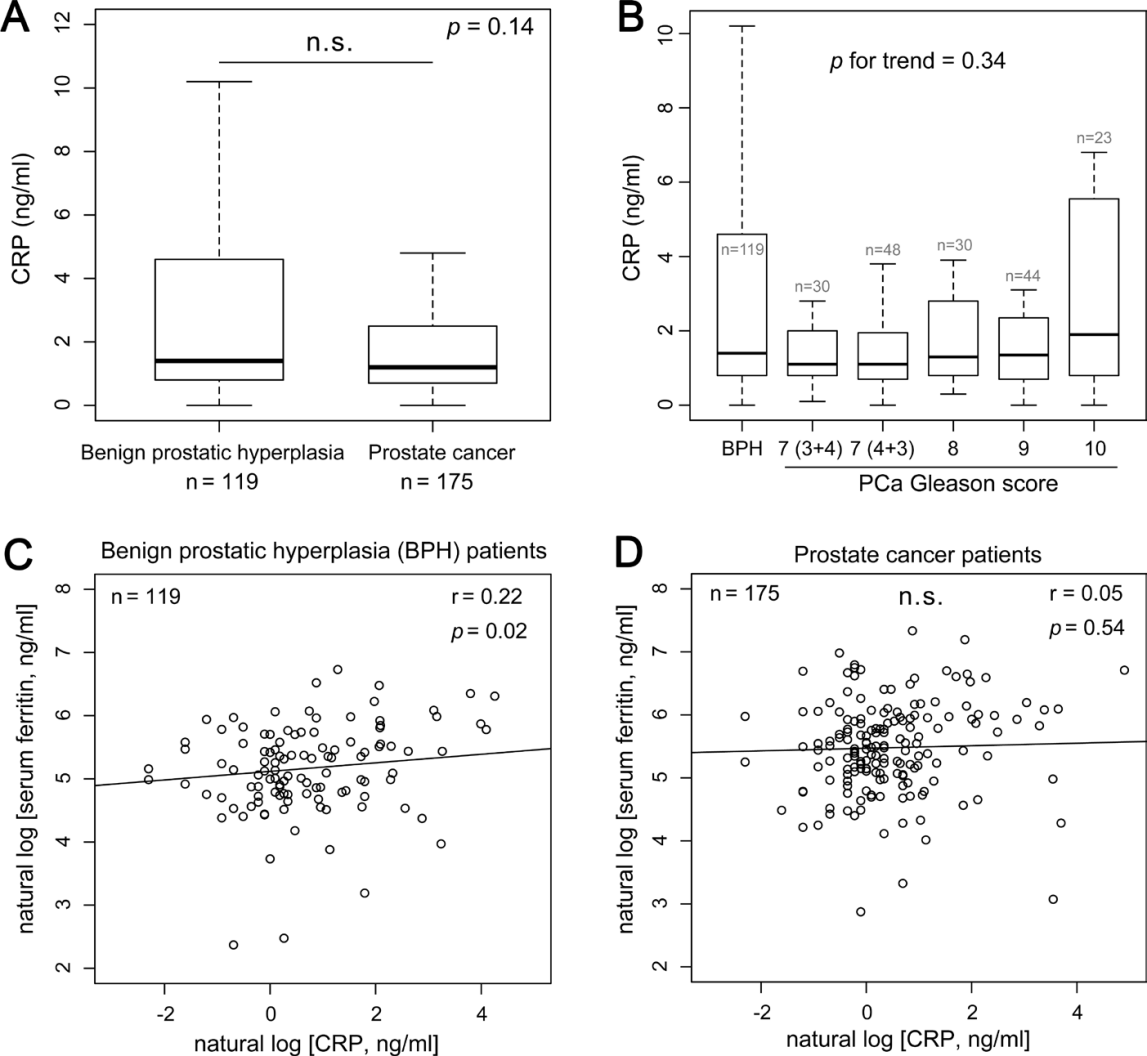
Serum ferritin levels are independent of the inflammation marker C-reactive protein (CRP) in prostate cancer patients (**A** and **B**) Box plots of CRP concentration measured in BPH patients (*n* = 119) and prostate cancer patients (*n* = 175) by group (A) and by disease severity (B). (**C** and **D**) Serum ferritin plotted against CRP for BPH patients (C) and prostate cancer patients (D).

Lastly, we measured the serum levels of the inflammation marker C-reactive protein (CRP) in 119 BPH patients and 175 prostate cancer patients. Our analysis showed that CRP levels did not differ significantly between the BPH and prostate cancer groups (Figure 4A). Moreover, in contrast to ferritin levels, we found no trend between CRP levels and disease severity (Figure 4B). Finally, although we found a weak but significant correlation between serum ferritin levels and CRP levels in the BPH group (Figure 4C), we found no correlation in the prostate cancer group (Figure 4D). Taken together, these results indicate that the increase in serum ferritin levels is not associated with inflammation in prostate cancer patients.

## DISCUSSION

In Asia, the incidence and mortality associated with prostate cancer is increasing [24]. Current diagnostic testing for prostate cancer relies primarily on measuring serum levels of total prostate-specific antigen (PSA), which can lead to the over-diagnosis and/or overtreatment of prostate cancer [25]. Therefore, we investigated the feasibility of using serum ferritin levels as a complementary non-invasive biomarker, thereby improving diagnostic specificity and clinical decision-making.

Increased levels of circulating ferritin, the body's primary iron storage protein, have been reported in a wide range of malignancies [13–16, 26–28]; moreover, increased ferritin levels are often related to decreased survival time and more aggressive disease progression [29]. For example, increased serum ferritin levels have been associated with the incidence, development, and metastasis of primary lung cancer [14]. Moreover, serum ferritin level has also been shown to be a reliable prognostic indicator in hepatocellular carcinoma [13] and other malignancies [12, 16, 22, 23, 30, 31]. Furthermore, serum ferritin measurements have been combined with more traditional cancer biomarkers such as CEA (carcinoembryonic antigen) and AFP (alpha-fetoprotein) for use as a diagnostic and/or prognostic marker in several types of cancer [14, 32]. On the other hand, and in contrast with our findings, Kuvibidila et al. reported an inverse correlation between serum ferritin levels and disease stage in prostate cancer patients [33]. Although the study by Kuvibidila *et al*. was limited to a relatively small cohort size of 34 patients with prostate cancer, the putative connection between serum ferritin levels and prostate cancer, as well as the diagnostic and prognostic value of serum ferritin, clearly warrants further investigation.

In our study, we measured circulating ferritin and PSA levels in 2002 patients with histologically confirmed prostate cancer and 951 patients with benign prostatic hyperplasia (BPH). We found high serum ferritin levels in the prostate cancer group; interestingly, nearly one-seventh of all prostate cancer patients had an abnormally high serum ferritin level (> 400 ng/ml). Moreover, this increased serum ferritin level was significantly correlated with both increased total PSA levels and increased prostate cancer risk. These results suggest that serum ferritin may serve as a possible diagnostic marker for prostate cancer. Our results also indicate that measuring serum ferritin and/or total PSA levels can provide even higher sensitivity when testing elderly patients (> 65 years of age) with hyperferritinemia (serum ferritin > 400 ng/ml), which suggests that serum ferritin and total PSA levels may be particularly valuable for diagnosing prostate cancer in elderly men.

To better understand the putative value of serum ferritin in evaluating prognosis in prostate cancer patients, we also analyzed the association between serum ferritin levels and currently used prognostic markers of prostate cancer. Interestingly, we found that a higher percentage of patients with advanced stage prostate cancer had high ferritin levels (> 400 ng/ml) compared with patients in relatively less advanced stages. Moreover, similar to our results with respect to diagnosing prostate cancer, we also found that measuring serum PSA levels provided more prognostic value in prostate cancer patients with hyperferritinemia. These results indicate that determining a prognosis may be more accurate among prostate cancer patients with high serum ferritin levels, which could help predict disease progression, thereby improving treatment efficacy.

Consistent with our findings with respect to serum ferritin, we also found that prostate tissue sections from prostate cancer patients had a higher percentage and distribution of increased ferritin levels compared with sections obtained from BPH patients. Moreover, we found a significant correlation between increased serum ferritin levels and increased tissue ferritin expression, which suggests a possible causal relationship between hyperferritinemia in prostate cancer patients and increased ferritin levels in tumor tissues. However, the precise mechanisms underlying increased serum ferritin levels in prostate cancer patients remain unclear.

Given that PSA alone may not provide sufficient sensitivity or specificity for diagnosing prostate cancer, Lilja *et al*. proposed the need for developing a feasible multivariable model [34]. Our results suggest that serum ferritin may provide the additional sensitivity and specificity needed in order to improve the diagnostic and prognostic value of the PSA test, particularly in patients with hyperferritinemia.

In conclusion, we systematically evaluated the diagnostic and prognostic value of using circulating ferritin as a non-invasive biomarker for prostate cancer in a large-scale case-control study. Our results provide compelling evidence to support the correlation between both circulating and tissue ferritin levels and prostate cancer risk. Moreover, our findings suggest a clear relationship between serum ferritin levels and the risk, diagnosis, and prognosis of prostate cancer. Taken together, our results indicate that serum ferritin could play a role in clinical practice, serving as a valuable prostate cancer biomarker to complement the standard PSA test.

## MATERIALS AND METHODS

### Patients

This case-control study was initiated in 2011 in order to investigate the relationship between serum ferritin levels and prostate cancer. From January 2011 through October 2015, data were collected from 2002 patients with histologically confirmed prostate cancer and from 951 patients with benign prostatic hyperplasia (BPH) at the First Affiliated Hospital of Zhejiang University (Hangzhou, China). The following inclusion criteria were used: no history of any other cancers, liver cirrhosis, or viral hepatitis, and no prior treatment for prostate cancer or BPH before blood collection. Tumor prognostic factors [35], including Gleason score, preoperative serum PSA, TNM (tumor, node, metastasis) classification, and percent positive biopsy cores, were recorded. The pathological stage was determined based on the 7th edition of the International Union Against Cancer (UICC) TNM classification. Percent positive biopsy cores refers to the ratio of the number of positive cores to the total number of biopsy cores, expressed as a percentage. The study was designed and performed in accordance with the Declaration of Helsinki, and the Ethics Committee of the First Affiliated Hospital of Zhejiang University approved this study.

### Serum parameters

Serum ferritin, serum total prostate-specific antigen (total PSA), and free prostate-specific antigen (free PSA) were measured using chemiluminescence immunoassays (ARCHITECT-i4000 immunology analyzer, Abbott Laboratories, Irving, TX). Lipid parameters, including triglycerides, total cholesterol, high-density lipoprotein (HDL) cholesterol, low-density lipoprotein (LDL) cholesterol, and very low-density lipoprotein (VLDL) cholesterol, were measured using a Hitachi Model 7600 Series Automatic Analyzer (Hitachi High-Technologies Corporation, Tokyo, Japan) in accordance with the manufacturer's instructions. This analyzer was also used to measure uric acid, fasting plasma glucose, and liver function parameters, including alanine transaminase (ALT), aspartate transaminase (AST), gamma-glutamyl transferase (GGT), direct bilirubin (DBIL), and indirect bilirubin (IBIL), using the respective reagents. Hemoglobin was measured using an XE2100 analyzer (Sysmex, Kobe, Japan). Serum CRP was measured using the latex agglutination method with an Abbott Aeroset automated analyzer. Blood pressure was measured using a mercury sphygmomanometer placed on the patient's right arm while at rest.

### Prostate tissue section immunohistochemistry

Sections were blocked with 10% goat serum for 20 minutes, and then incubated with anti-ferritin heavy chain antibody (Catalog #EPR3005Y; 1:300; Abcam, Cambridge, UK) for 90 minutes at 37°C. The slides were then incubated with the appropriate secondary antibody (Dako Real Envision/HRP, Rabbit/Mouse, K5007) and visualized using 3,3′-diaminobenzidine with peroxidase substrate (Dako Real DAB Chromogen, K5007). The sections were counterstained with hematoxylin. Ferritin immunostaining was semi-quantitatively scored using a composite score obtained by multiplying the mean staining intensity value by the percentage of positively stained cells. Staining intensity was classified as absent (0), weakly positive (1), moderately positive (2), or strongly positive (3).

### Statistical analyses

All summary data in the tables are presented as the mean (with standard deviation) or median (with interquartile range). *P*-values between two-groups were obtained using the Wilcoxon rank-sum test. The Pearson's chi-square test was used to analyze categorical data. A linear regression model was used to analyze the association between serum parameters and serum PSA levels, as well as the association between serum parameters and prostate cancer risk. In the regression analysis, serum total PSA, free PSA, free PSA/total PSA ratio, and triglyceride levels were natural log-transformed to avoid a skewed distribution. All statistical analyses were performed using R software. Receiver operating characteristic (ROC) curves were generated and calculated using the pROC package in R [36]. We used the Jonckheere-Terpstra test (clinfun package in R) to determine the *p*-value for the trend in serum parameter levels based on the Gleason score. In the box plots, the whiskers represent the 1.5 interquartile range. Differences with *p* < 0.05 were considered statistically significant.
